# Pyroglutamate and Isoaspartate modified Amyloid-Beta in ageing and Alzheimer’s disease

**DOI:** 10.1186/s40478-017-0505-x

**Published:** 2018-01-03

**Authors:** Maria Luisa Moro, Andrew Stephen Phillips, Katie Gaimster, Christian Paul, Amritpal Mudher, James A. R. Nicoll, Delphine Boche

**Affiliations:** 1 Clinical Neurosciences, Clinical and Experimental Sciences Academic Unit, Faculty of Medicine, University of Southampton, Southampton, UK; 2 Centre for Biological Sciences, Faculty of Natural & Environmental Sciences, University of Southampton, Southampton, UK

**Keywords:** IsoAspartate, Pyroglutamate, Alzheimer’s disease, Amyloid-beta, Ageing

## Abstract

Alzheimer’s disease (AD) is the most common cause of dementia among older adults. Accumulation of amyloid-β (Aβ) in the brain is considered central in AD pathogenesis and its understanding crucial for developing new diagnostic and therapeutic approaches. Recent literature suggests that ageing may induce post translational modifications in Aβ, in the form of spontaneous amino acid modifications, which enhance its pathogenic properties, contributing to its aggregation.

In this study, we have investigated whether the isoaspartate (IsoD-Aβ) and pyroglutamate (pE3-Aβ) modified forms of Aβ are significantly associated with AD pathology or represent markers of ageing. Cerebral neocortex of 27 AD cases, 32 old controls (OC) and 11 young controls (YC) was immunostained for pE3-Aβ and IsoD-Aβ, quantified as protein load and correlated with other Aβ forms and p-TAU. IsoD-Aβ and pE3-Aβ were detected at low levels in non-demented controls, and significantly increased in AD (*p* ≤ 0.001), with a characteristic deposition of IsoD-Aβ in blood vessel walls and pE3-Aβ within neurons. Both AD and OC showed positive associations between IsoD-Aβ and Aβ (*p* = 0.003 in AD and *p* = 0.001 in OC) and between IsoD-Aβ and pE3-Aβ (*p* = 0.001 in AD and OC). This last association was the only significant pE3-Aβ correlation identified in AD, whereas in the control cohorts pE3-Aβ also correlated with Aβ and AβPP (*p* = 0.001 in OC and *p* = 0.010 in YC).

Our analyses suggest that IsoD-Aβ accumulation starts with ageing; whereas pE3-Aβ deposition is more closely linked to AD. Our findings support the importance of age-related modifications of Aβ in AD pathogenesis.

**Electronic supplementary material:**

The online version of this article (10.1186/s40478-017-0505-x) contains supplementary material, which is available to authorized users.

## Introduction

Alzheimer’s disease (AD) is the most common cause of dementia in the elderly population. The molecular pathogenesis of this disease is only partially understood and the comprehension of new key aspects of its neurobiology is crucial for the future development of early diagnostic approaches and effective treatments [34]. AD is characterized by the intra- [5, 39] and extracellular accumulation in the brain of aggregated amyloid-β (Aβ), and of hyperphosphorylated tau (p-TAU) within the neurons [32]. Aβ misfolding, resulting in its aggregation into oligomers, protofibrils and fibrils, is considered crucial in the development of AD [33] and synergistic with tau pathology [24]. Aβ is a family of peptides generated by β- and γ-secretase sequential cleavages of the amyloid precursor protein (APP) and currently regarded as one of the major therapeutic targets in AD [34].

Ageing is considered the main risk factor for AD, but the pathway through which ageing contributes to Aβ misfolding remains unclear. In particular, we still do not know whether ageing induces molecular changes in Aβ, driving its accumulation in the brain. Recent literature suggests that ageing may induce post-translational modifications, in particular spontaneous Aβ amino acid modifications, defined as age-related modifications, which enhance its pathogenic properties [9, 21, 23, 27, 44], leading to the “Protein ageing Hypothesis of AD” [23]. It is conceivable that accumulation of age-related amino acid modifications could start early in the ageing process [21]. Consequently, the understanding of the relationship between age-related-modifications of Aβ and AD pathology could potentially identify new diagnostic and therapeutic approaches in early AD.

Prominent examples of these age-modified forms of Aβ include isomerization (isoD-Aβ) and racemization of aspartate residues, and pyroglutamate formation at the N-terminal of Aβ (pE-Aβ) [14]. IsoD-Aβ, similarly to the racemized form of aspartate, is the result of a chemically spontaneous and non-enzymatic reaction that introduces an additional methylene group in the peptide backbone of Aβ [37]. The formation of pE-Aβ is the consequence of a truncation at the level of a N-terminal glutamate, followed by the dehydration catalyzed by Glutaminyl Cyclase to form the cyclic pyroglutamate [11]. Evidence supports a direct role of these modifications in altering the intrinsic properties of Aβ, as to accelerate its deposition, or to impair its clearance and degradation [23, 37]. In vitro studies have shown that IsoD-Aβ was associated with accelerated Aβ aggregation and fibril formation [23, 36]; and known mutations, where aspartic acid of Aβ is replaced by asparagine and then modified into isoD, are associated with early-onset AD and high levels of Aβ deposition [3, 6, 42]. Similar observations were reported for pE-Aβ, in particular with the modification at the glutamate in position 3 of Aβ (pE3-Aβ). It is toxic in primary culture of neurons and astrocytes [28], and its expression in mouse and *Drosophila* brains acts as an important source of toxicity, displaying an accelerated aggregation, enhanced synaptic toxicity, high stability and resistance to degradation [19, 28, 31, 38]. Moreover, it was demonstrated that small amounts of pE-Aβ oligomers are sufficient to trigger the aggregation of unmodified Aβ1–42, leading to the formation of hypertoxic Aβ1–42 oligomers [22]. Of note, passive immunization with a pE-Aβ monoclonal antibody in APPswe/PS1ΔE9 AD mouse model, was able to lower Aβ plaque burden and prevent cognitive impairment [4]. Therefore, the formation of pE- and IsoD-Aβ may have a role in the pathological process of Aβ aggregation and accumulation.

In this study, we have addressed the question whether these Aβ modifications are significantly associated with AD pathology or if they represent physiological markers of ageing, using *post-mortem* brain tissue from AD cases compared to non-neuropathological old and young controls.

## Materials and methods

### Case selection

Seventy *post-mortem* cases were investigated divided among 3 cohorts as follows: 27 AD cases, 11 young controls defined as with no significant neuropathological abnormality (YC, <63 years old) and 32 old control cases with no significant neuropathological abnormality (OC, ≥ 63 years old), age-matched with the AD cohort. A summary of the cohorts is presented in Table 1 with additional information available in Additional file 1: Table S1. All AD cases had a clinical diagnosis of probable Alzheimer’s disease according to NINCDS–ADRDA criteria and cases with concomitant pathology were excluded. Diagnosis was made during life by an experienced clinician and post-mortem neuropathological consensus criteria for AD were satisfied, including Braak stage, by an experienced neuropathologist.

**Table 1:** Summary of the AD, old and young cohorts

Case	Gender	Age at death	APOE status	Braak stage	Dementia duration (years)
AD	15F:12M	63–88	20ε4+: 5ε4^_^	IV-VI	3–17
OC (*n* = 32)	16F:16M	64–97	5ε4+: 27ε4^_^	0-III	n/a
YC (*n* = 11)	6F:5M	26–62	n/d	n/d	n/a

*n/a* non-applicable

*n/d* non-determined

### Immunohistochemistry

**Fig. 1:**
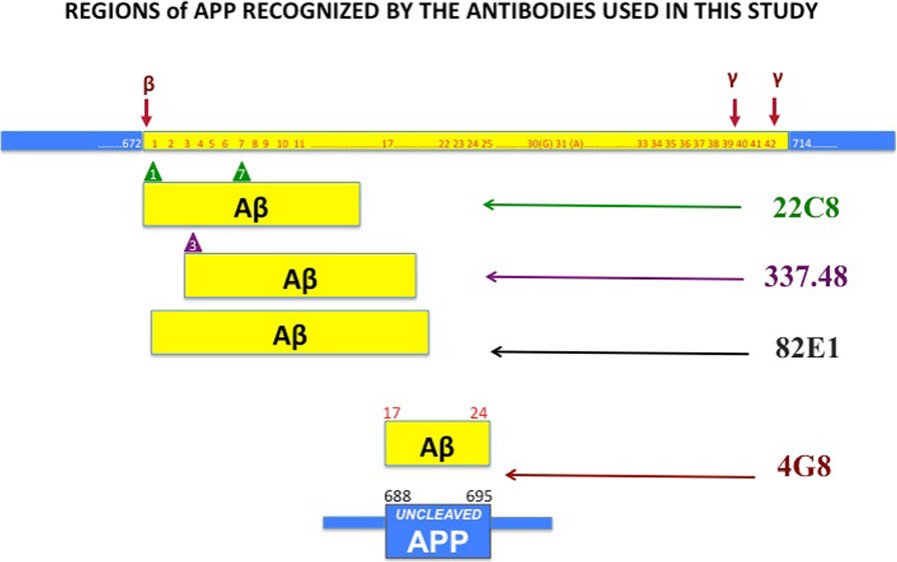
Regions of APP recognized by the antibodies used in this study. Top of the figure: Illustration of APP. Aβ region is labelled in yellow and β and γ-secretase cleavage sites are indicated in red. Bottom of the figure: Illustration of the Aβ and APP regions recognized by 22C8, 337.48, 82E1, 4G8 clones. The positions of IsoD- and pE3**-**Aβ modifications recognized, respectively by clones 22C8 and 337.48, are indicated with triangles

Four μm formalin-fixed paraffin-embedded sections from the inferior parietal lobule (Brodmann area 40) were retrieved from the Brain banks for all cases. Protocols for tissue fixation and processing were similar in both brain banks. In addition, the staining was performed in batches with each batch including cases from all 3 cohorts to ensure compatibility of the staining. The following primary mouse monoclonal antibodies were used: 22C8 against 1–12 Aβ with 1,7 IsoAspartate modification (IsoD-Aβ) provided by Elan Pharmaceuticals Inc., US [29, 30]; 337.48 specific to Aβ with pyroglutamate at the third glutamate position (pE3-Aβ, BioLegend, US); 4G8, specific for the amino acid residues 17–24 of Aβ, which reacts to the abnormally processed isoforms, as well as precursor forms (AβPP) [1, 2, 12](BioLegend, US); 82E1, an Aβ N-terminal specific antibody, which does not cross react with non-β secretase cleaved APP (IBL, Japan, [8]); AT8, directed against the human tau, phosphorylated at Ser202 and Thr205 (p-TAU) (ThermoFisher Scientific, US) (Fig. 1 and Table 2).

**Table 2:** Antibodies and immunohistochemistry conditions

Clone	Specificity	Company	Modified amino acid	Antigen Abbreviation	Dilution	Antigen retrieval method
22C8	IsoAspartate Aβ	Elan Pharmaceuticals	Aspartate in position 1,7	IsoD-Aβ	1:10,000	Formic acid 80% for 30 min
337.48	pyroglutamate Aβ	BioLegend	Glutamate in position 3	pE3-Aβ	1:100	Formic acid 100% for 3 min + EDTA buffer and microwave
4G8	Aβ and APP	BioLegend	n/a	AβPP	1:2000	Formic acid 80% for 30 min
82E1	N-terminus end specific	IBL	n/a	Aβ	1:100	Formic acid 80% for 30 min
AT8	Phosphorylated tau	ThermoFisher Scientific	Ser202 and Thr205	p-TAU	1:500	Sodium citrate buffer and pressure cooker

Immunohistochemistry was performed using the appropriate antigen retrieval method (Table 2). Biotinylated secondary antibody (rabbit anti-mouse) was from Dako (Denmark), and normal serum and avidin–biotin complex from Vector Laboratories (UK). Bound antibody was visualized using the avidin–biotin–peroxidase complex method (Vectastain Elite ABC) with 3,3′-diaminobenzidine as chromogen and 0.05% hydrogen peroxide as substrate (both from Vector Laboratories, UK). All sections were dehydrated before mounting in DePeX (VWR International, UK). Sections incubated in the absence of the primary antibody were included as negative controls.

### IsoD-Aβ and pE3-Aβ quantification

Quantification was performed blind to the experimental group and identity of the cases. For each antibody and case, 30 images of cortical grey matter were taken using a ×20 objective lens, in a zigzag sequence in order to ensure that all cortical layers were represented in the quantification. The sampling pattern between all cases was consistent, starting at the depth of the sulcus and progressing up the sulcal wall to the gyral surface. The acquired images were analysed using ImageJ version 1.49 software (developed by Wayne Rasband NIH, US) with a threshold applied to the image to select and measure the total amount of specific immunostaining. The same threshold setting was maintained for all images of all cases stained for the same antibody, and the area fraction of the measure function provided the proportion (%) of the stained area related to the total area of the image (expressed as protein load).

### Semi quantitative assessment

Immunodetection for Iso-DAβ and pE3-Aβ was assessed as present or absent according to the staining being defined as: intraneuronal deposits [5], dense-core plaques, diffuse plaques and vessel wall deposits (i.e. cerebral amyloid angiopathy: CAA) [35]. The staining was independently reviewed by two operators.

### Statistical analysis

To compare the protein load of the different Aβ forms and p-TAU between the cohorts, the normality of each marker was assessed through examination of quantile-quantile plots (not shown). As the data were non-parametric, the Kruskal-Wallis test was performed for comparison among the three groups for each marker. To assess whether the presence of pE3-Aβ and IsoD-Aβ immunostaining in intraneuronal deposits, dense-core plaques, diffuse plaques and CAA differed significantly between AD and OC and OC and YC, the Fisher’s Exact Test was used.

To investigate the relationship of isoD-Aβ and pE3-Aβ with the other Aβ forms and p-TAU, correlations were performed using the Pearson or Spearman correlation coefficients, as determined by the normality of the markers. The threshold for statistical significance was set at 5% for intergroup comparisons and Fisher’s test and 1% for correlations, as determined by the use of SPSS 21.0 (IBM, US).

## Results

### Quantification of IsoD and pE3-Aβ

**Fig. 2:**
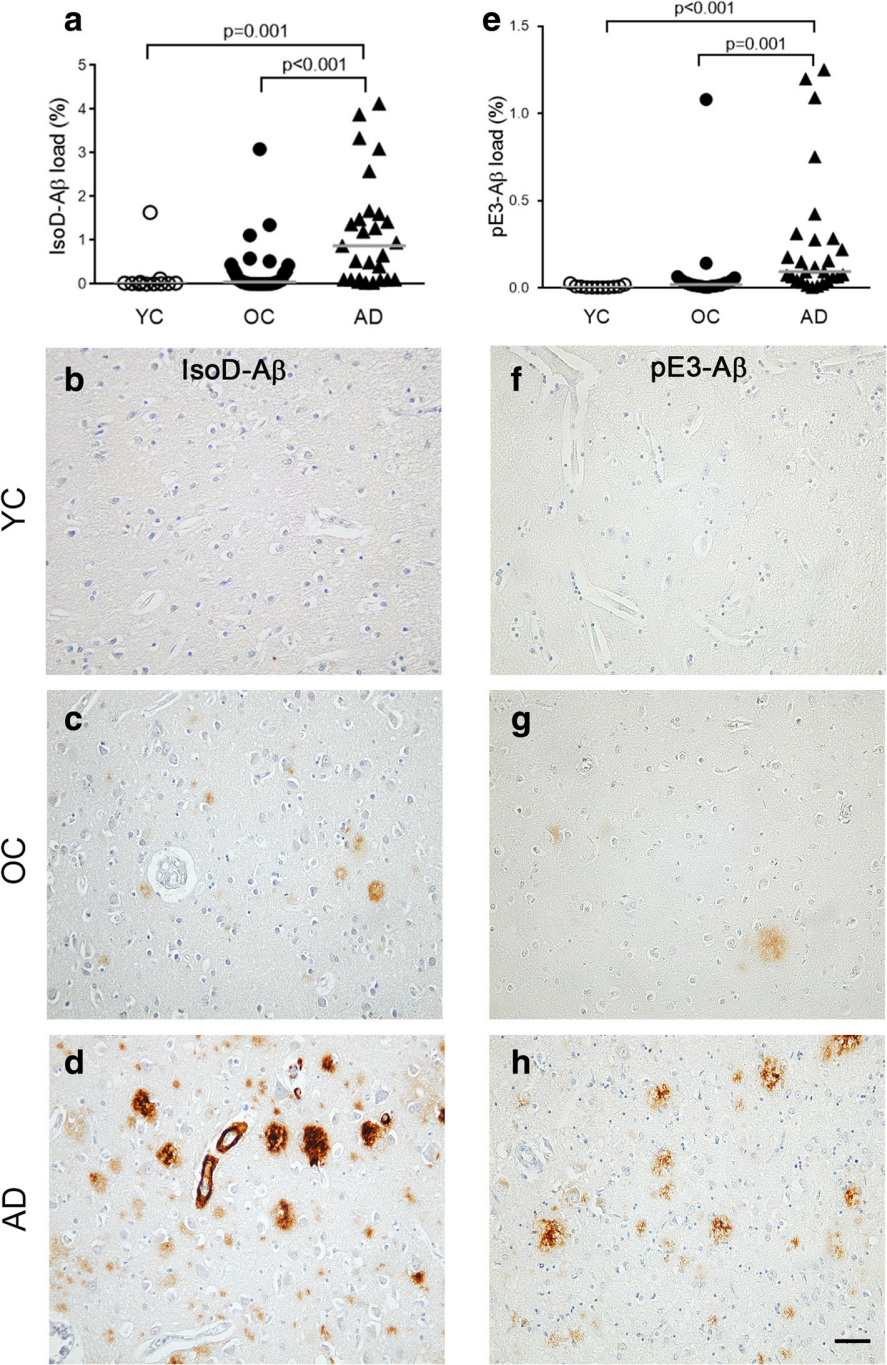
Quantification of IsoD-Aβ and pE3-Aβ loads. Both IsoD-Aβ (**a**) and pE3-Aβ (**e**) loads are significantly higher in the AD cases than in the OC and YC groups. Illustrations of immunostaining for IsoD-Aβ (**b-d**) and pE3-Aβ (**f-h**) in the 3 cohorts. Scale bar = 50 μm

The quantification of IsoD-Aβ, independently of the location of the staining, revealed that IsoD-Aβ load was significantly higher in AD compared to OC (*p* < 0.001) and AD vs YC (*p* = 0.001) cohorts. No significant difference was identified between OC and YC groups (Fig. 2a).

Similarly, the pE3-Aβ load was significantly higher in AD vs OC (*p* = 0.001) and AD vs YC (*p* < 0.001) cases; while no significant difference was found between OC and YC groups (Fig. 2e).

### IsoD-Aβ and pE3-Aβ immunodetection and locations

**Fig. 3:**
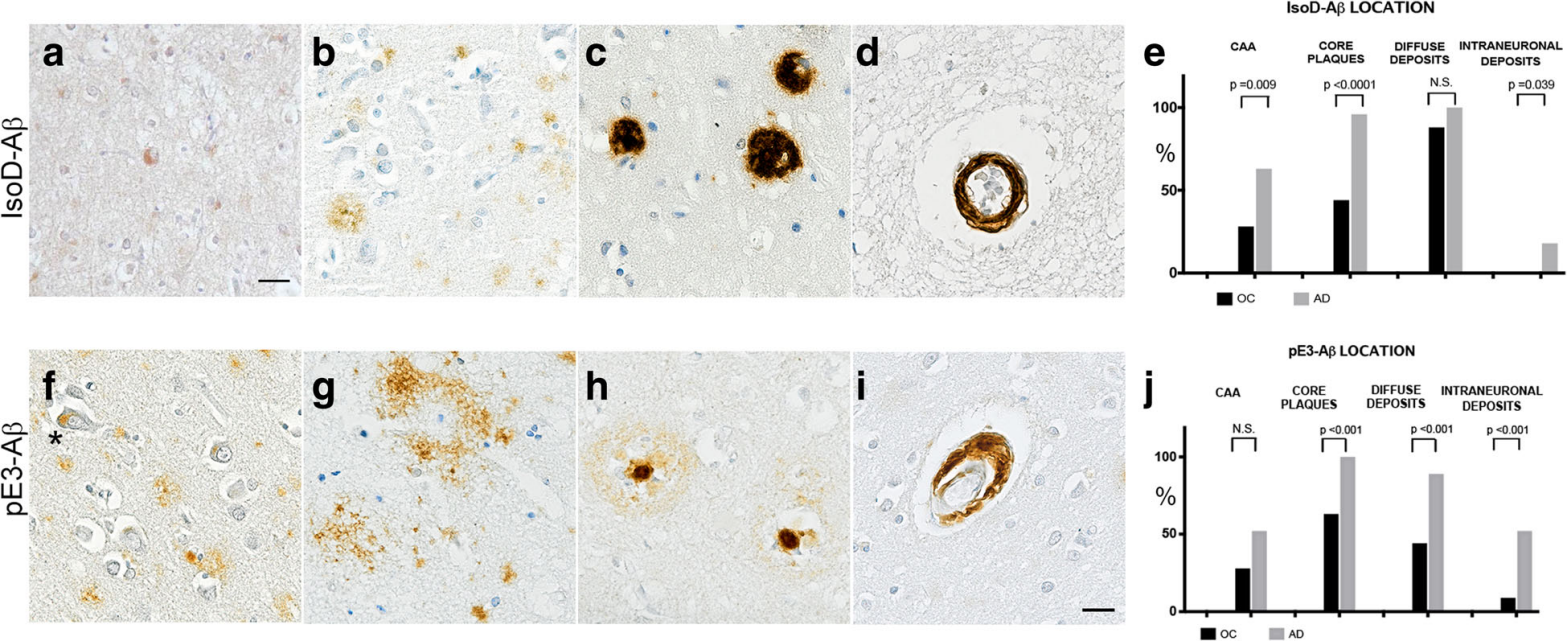
Location of IsoD-Aβ and pE3-Aβ immunostaining. Illustrations of the different neuropathological features detected by immunostaining for IsoD-Aβ (upper panel) and pE3-Aβ (lower panel): intraneuronal (**a**, f*), diffuse deposits (**b, g**), core plaques (**c, h**), and cerebral amyloid angiopathy (CAA) (**d, i**). The assessment of the different neuropathological features shows that: (**e**) IsoD-Aβ in CAA, core plaques and neurons was significantly more frequent in AD vs OC; however intraneuronal IsoD-Aβ was limited to only 18% of AD cases; (**j**) pE3-Aβ was significantly more frequent in neurons, diffuse deposits and core plaques in AD compared to OC cases. Scale bar (**a**) = 50 μm; (**b-i**) = 25 μm

IsoD-Aβ and pE3-Aβ immunostaining was observed in the commonly identified Aβ locations in the brain: intraneuronal deposits, diffuse deposits, dense-core plaques, and CAA (Fig. 3a-d and f-i). However significant differences were observed between OC and AD regarding the presence of some of these neuropathological features (Fig. 3e-j).

Overall, the comparison between AD and OC cases revealed that IsoD-Aβ immunostaining in core plaques and CAA was significantly more frequent in the AD vs OC cases (core plaques: AD = 96% vs OC = 44%, *p* < 0.0001; CAA: AD = 63% vs OC = 28%, *p* = 0.009) (Fig. 3e). IsoD-Aβ intraneuronal deposits were also significantly more present in AD than OC; however this feature was limited to only a minority of AD cases (AD = 18%; OC = 0%, *p* = 0.039). No significant difference was observed instead in relation to IsoD-Aβ stained diffuse deposits (*p* = 0.118) (Fig. 3e).

No IsoD-Aβ staining was identified in YC (Fig. 5j), with the exception of 2 cases among the oldest, who were 59 and 62 years old and had some diffuse and core plaques (Fig. 5l).

pE3-Aβ immunostaining was significantly more present in AD vs OC cases in intraneuronal deposits (AD = 52% vs OC = 9%, *p* < 0.001); in diffuse plaques (AD = 89% vs OC = 44%, *p* < 0.001); and core plaques (AD = 100% vs OC = 63%, *p* < 0.001) (Fig. 3j). No significant difference was observed instead in relation to pE3-Aβ CAA (*p* = 0.065).

pE3-Aβ was negative in the majority of YC (Fig. 5k). Only two YC cases, the same cases which have sparse isoD-Aβ deposits, also had sparse pE3-Aβ-positive immunostaining mainly as diffuse plaques, with the case aged 62 also having few core plaques (Fig. 5m).

### Relationship between IsoD, pE3-Aβ and hallmarks of AD

**Fig. 4:**
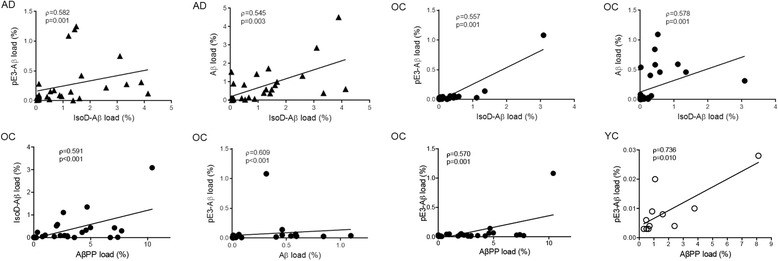
Correlation analysis. Representation of the most significant correlations (*p* < 0.01) observed in the AD, OC and YC cohorts

**Fig. 5:**
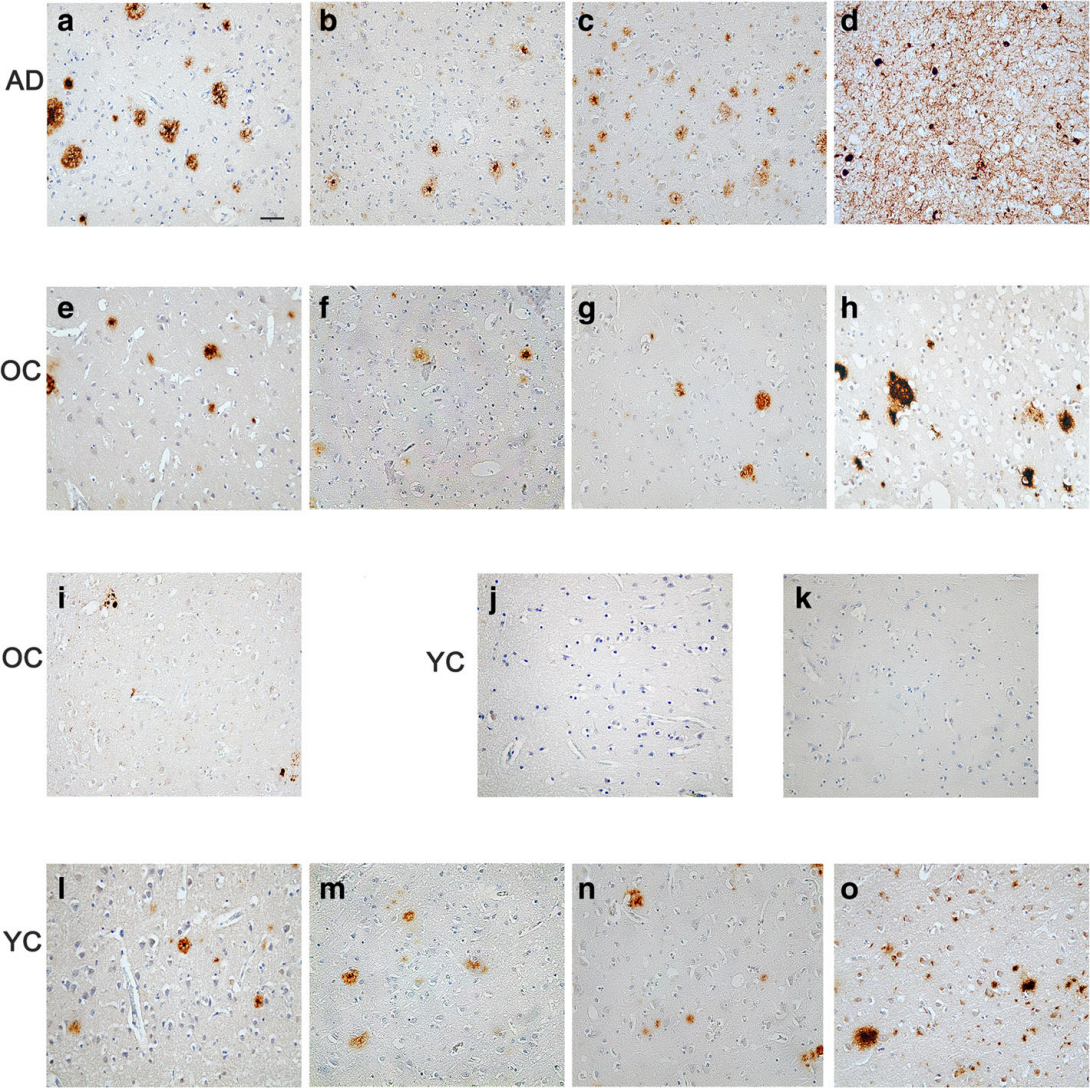
Pictures of IsoD-Aβ, pE-Aβ, Aβ, AβPP and p-TAU in AD, OC, and YC. Representative pictures of IsoD-Aβ (**a**), pE3-Aβ (**b**), Aβ (**c**), p-TAU (**d**) staining of AD cases, the significant correlations of which are described in Table 3. Representative pictures of IsoD-Aβ (**e**), pE3-Aβ (**f**), Aβ (**g**), AβPP (**h**) and p-TAU (**i**) staining of OC cases, the significant correlations of which are described in Table 3. IsoD-Aβ (**j**) and pE3-Aβ (**k**), as well as Aβ, AβPP and p-TAU staining were negative in the majority of YC. Only in two YC cases, IsoD-Aβ (**l**), pE3-Aβ (**m**), Aβ (**n**), AβPP (**o**) deposits were detectable. Scale bar = 50 μm

Correlations were performed in the 3 cohorts between IsoD and pE3-Aβ loads each other, and respectively, with AβPP (clone 4G8), Aβ (clone 82E1), and p-TAU (AT8 antibody). All analyses are presented in Table 3 and Fig. 4 and the representative pictures in Fig. 5. In the AD group, two significant positive correlations were found for IsoD-Aβ with pE3-Aβ (ρ = 0.582, *p* = 0.001) and Aβ (ρ = 0.545, *p* = 0.003). In the OC cohort, significant positive associations were identified for IsoD-Aβ with pE3-Aβ (ρ = 0.557, p = 0.001), Aβ (ρ = 0.578, p = 0.001) and AβPP (ρ = 0.591, *p* < 0.001).

**Table 3:** Results of correlation analyses within the 3 cohorts

	AD cases				OC cases				YC cases			
	pE3-Aβ	AβPP	Aβ	p-TAU	pE3-Aβ	AβPP	Aβ	p-TAU	pE3-Aβ	AβPP	Aβ	p-TAU
IsoD-Aβ	**ρ = 0.582**	ρ = −0.026	**ρ = 0.545**	ρ = 0.336	**ρ = 0.557**	**ρ** = **0.591**	**ρ** = **0.578**	ρ = 0.289	ρ = 0.097	ρ = 0.145	ρ = 0.247	ρ = 0.382
	**p = 0.001**	p = 0.897	**p = 0.003**	p = 0.086	**p = 0.001**	**p < 0.001**	**p = 0.001**	p = 0.115	p = 0.778	p = 0.670	p = 0.465	p = 0.246
pE3-Aβ		ρ = 0.119	ρ = 0.098	ρ = 0.389		***ρ = 0.570***	**ρ = 0.609**	ρ = 0.380		**ρ = 0.736**	ρ = 0.663	ρ = 0.107
		p = 0.554	p = 0.628	p = 0.045		***p = 0.001***	**p < 0.001**	p = 0.038		**p = 0.010**	p = 0.026	p = 0.755

bold** correlation significant at the 0.01 level (2-tailed)

italic: Pearson’s ρ and p-value

non-italic: Spearman’s ρ and p-value

For pE3-Aβ in OC cohort, in addition to the correlation identified with IsoD-Aβ, there were positive significant associations with Aβ (ρ = 0.609, p < 0.001) and AβPP (ρ = 0.570, *p* = 0.001). Within the YC group, a positive correlation was observed between AβPP and pE3-Aβ load (ρ = 0.736, *p* = 0.010).

## Discussion

In this study, we have investigated two post-translational modifications of Aβ, IsoD-Aβ and pE3-Aβ, normally defined as age-related modifications [11, 21, 25], and explore whether their accumulation is significantly increased in AD patients compared with age-matched controls and younger controls. Our findings show that both IsoD-Aβ and pE3-Aβ can be detected at a low level in non-demented age-matched controls but both are at significantly higher levels in AD.

It is possible that these modified forms of Aβ start to form and aggregate years before other molecular lesions and symptoms of disease clearly appear. Indeed pE3-Aβ has been detected in the past not only in plaques but also in dispersible and soluble Aβ aggregates outside the plaques and described in pathologically preclinical AD cases [26]. Consequently in our study, some of non-demented age-matched controls might have actually died after IsoD-Aβ and pE3-Aβ accumulation in the brain had started, but before the formation of a significant Aβ burden.

The higher level of IsoD-Aβ in AD may be explained by the properties of the amino acid of Aβ. There is evidence that IsoD forms most easily at sequences in which the side chain of the C-flanking amino acid is relatively small and hydrophilic [36]. The most favourable C-flanking amino acids are Glycine (G), Serine (S), and Histidine (H). The Aβ sequence has S and G residues at the C-term of aspartate 7 (D7), rendering this residue most likely to be spontaneously transformed to IsoD with ageing. The other aspartate residue analysed, D1, is the first amino acid of the Aβ sequence, which is also the β-secretase site of APP. D1 does not have C-term residues favourable to spontaneous isomerization. However, its isomerization in AD can be driven by other chemical conditions, e.g. oxidative stress and production of radicals [18] as demonstrated for D-racemization [13, 40], occurring through the same chemical intermediate of D-isomerization [21]. Therefore, it is reasonable to hypothesize that in AD, IsoD-Aβ accumulates at a significantly higher level than during ageing due to faster isomerization of D7 or to a possible additional modification of D1; processes that are likely not to be mutually exclusive.

The increased pE3-Aβ in AD may be explained by increased expression of Glutaminyl cyclase (QC), the enzyme that catalyses the conversion of glutamate into pyroglutamate. QC was found in the cortex of AD patients [20] and its levels correlated with insoluble pE3-Aβ aggregates, and not with unmodified Aβ peptides. Noteworthy, in the same patients, the insoluble pE3-Aβ correlated with the cognitive decline better than elevated level of unmodified Aβ [20].

As expected, in our study, the majority of YC cases did not have IsoD- or pE3-Aβ forms. Of note, the only cases that had some immunostaining were among the oldest, aged 59 and 62 years, and thus close to the old control group.

Similar observations were reported in a study focused on brain tissues of cases with Down Syndrome (DS). Trisomy 21 is associated with the progressive development of AD neuropathology as observed in DS people in their forties. A study reported that deposits containing pE3-Aβ were not detectable in DS cases under 27 years old, while they were present in older cases [16].

Interestingly, our assessment of different features of Aβ deposits shows that the differences between AD and controls are not only quantitative, but also related to the specific compartments of brain parenchyma and vasculature where IsoD-Aβ and pE3-Aβ accumulate. IsoD-Aβ was largely extracellular, in core plaques as well as in blood vessel walls, in accordance with previous published data [14, 36]. While the majority of AD cases had both these features, they were less prevalent in the OC group. In AD, IsoD-Aβ in core plaques and CAA was more prevalent than IsoD-Aβ within neurons. This is in agreement with the results of a previous study focused on DS and small group of AD and OC with IsoD-Aβ detected in amyloid cores and vascular amyloid [10]. The limited intraneuronal detection of IsoD-Aβ is consistent with the location in the cytoplasm and endoplasmatic reticulum of L-isoaspartyl methyltransferase, the enzyme able to repair IsoD to L-aspartyl residue. This explains that possibilities to repair extracellular IsoD are very limited [36], implying that once extracellular IsoD-Aβ deposits are formed, differently from intraneuronal deposits, they might likely remain unrepaired.

We detected pE3-Aβ in the vasculature, as previously reported [17, 41], but in our larger cohorts the prevalence of this feature was not significantly greater in AD than controls. However, pE3-Aβ in AD was found in diffuse deposits, core plaques and intraneuronal deposits, in agreement with previous studies [17, 45] and in these specific locations, pE3-Aβ was significantly more frequent in AD than OC. Of note, pE3-Aβ in core plaques was the only feature observed in the majority of both AD and OC. A larger difference in the comparison of pE3-Aβ neuropathological features between OC and AD was related to intraneuronal pE3-Aβ, which was limited to few OC cases while present in 52% of AD. This suggests that intraneuronal accumulation of pE3-Aβ might be characteristic of AD, compatible with an increased somatic activity of QC enzyme. The presence of pE3-Aβ in plaques, might result instead from the possibility of secretion of this enzyme into the extracellular space, as observed in some experimental studies [7].

Our correlation analyses showed a number of relationships between IsoD and pE3-Aβ and hallmarks of AD. We observed positive associations of IsoD-Aβ with pE3-Aβ and Aβ (peptide with no N-terminal modifications) in AD. Of note, the same relationships were found in OC, although they had less IsoD-Aβ. This suggests that the presence of IsoD is associated with a process of accumulation of Aβ forms, already in progress with ageing. This is indeed in agreement with studies showing that isoaspartate post-translational modifications affect the fibrillization and toxicity properties of Aβ [3], possibly with a role in seeding the accumulation of Aβ species [43]. Interestingly in OC, both IsoD- and pE3-Aβ showed associations with AβPP and Aβ loads. In AD, despite the increased accumulation of IsoD-Aβ and pE3-Aβ, there was no association of the two modified forms with AβPP and only IsoD-Aβ correlated with Aβ load, while pE3-Aβ was independent from it. This may imply that different mechanisms control the accumulation of these Aβ modifications in ageing and in AD, with the existence of a direct link between IsoD-/pE3-Aβ accumulation and AβPP only during ageing.

p-TAU was associated with pE3-Aβ in both AD and OC cohorts, but not with the same strength as the other described associations. This relationship was already described in a study performed in 18 AD and 23 age-matched controls [17], where the data of both cohorts were combined. Accordingly, when we pooled AD and OC data, our association became stronger (ρ=0.6704, *p* < 0.0001) and similar to the previous published study, as shown in Additional file 1: Figure S1. Therefore, it is reasonable to hypothesize that the presence of pE3-Aβ in the brain leads to further neuropathological lesions, like the hyperphosphorylation of tau.

In summary, we have observed that high levels of IsoD- and pE3-Aβ are present in AD brain. IsoD-Aβ accumulation appears to be mainly related to ageing and, both in old and AD brains, this modification is associated with the build-up of unmodified Aβ as well as pE3-Aβ. In AD, IsoD-Aβ load is significantly higher than in OC and also accumulates significantly more in blood vessel walls. In contrast, pE3-Aβ appears to be more specifically linked to the disease process. In AD brain, pE3-Aβ deposition is significantly higher than in controls with a characteristic location in neurons. However, both post-translational modifications cannot be related at the moment to any temporal sequences of events.

## Conclusions

These findings underline the importance of age-related post-translational modifications of Aβ in relation to AD. They complement the studies that have revealed the complexity of Aβ chemistry and activity [3, 15]. In addition, our observations support the relevance of investigating IsoD-Aβ and pE3-Aβ levels as potential biomarkers for AD and the impact of the different Aβ-targeted therapies on these modified forms of Aβ. This research could benefit in the future from the development and optimization of new analytical methods [46], able to separate with high resolution the different forms of Aβ peptides in biological fluids.

## Additional file

Additional file 1: Contains all supplemental information mentioned in this manuscript. **Table S1.** Characteristics of the old control and AD cohorts. **Figure S1.** Representation of the correlations between pE3-Aβ and phospho-TAU in OC and AD groups together. (DOC 203 kb)

## Abbreviations

AD: Alzheimer’s disease
APP: the amyloid precursor protein
Aβ: Amyloid-beta
AβPP: Precursor forms and Aβ
CAA: Cerebral amyloid angiopathy
D: Aspartate
E: Glutamate
G: Glycine
H: Histidine
IsoD-Aβ: Isoaspartate modified amyloid-beta
OC: Old controls
pE-Aβ: Pyroglutamate modified amyloid-beta
p-TAU: Hyperphosphorylated tau
QC: Glutaminyl cyclase
S: Serine
YC: Young controls
