# Supplementary material for: Pyroglutamate and Isoaspartate modified Amyloid-Beta in ageing and Alzheimer’s disease
Source: Acta Neuropathol Commun. 2018 Jan 3;6:3. doi: 10.1186/s40478-017-0505-x (PMC5753481; doi:10.1186/s40478-017-0505-x)
Supplement: Additional file 1: — Contains all supplemental information mentioned in this manuscript. Table S1. Characteristics of the old control and AD cohorts. Figure S1. Representation of the correlations between pE3-Aβ and phospho-TAU in OC and AD groups together. (DOC 203 kb) [file 40478_2017_505_MOESM1_ESM.doc]

**ADDITIONAL FILE 1**

**Supplementary Material**

**PYROGLUTAMATE AND ISOASPARTATE MODIFIED AMYLOID-BETA IN AGEING AND ALZHEIMER’S DISEASE**

*Acta Neuropathologica Communications*

Maria Luisa Moro^1#^, Andrew Stephen Phillips^1^, Katie Gaimster^1^, Christian Paul^1^, Amritpal Mudher^2^, James AR Nicoll^1^, Delphine Boche^1#^

^1^Clinical Neurosciences, Clinical and Experimental Sciences, Faculty of Medicine, University of Southampton, Southampton UK

^2^Centre for Biological Sciences, Faculty of Natural & Environmental Sciences, University of Southampton, Southampton, UK

#Correspondence should be addressed to Maria Luisa Moro or Delphine Boche

Clinical Neurosciences, Clinical and Experimental Sciences, Faculty of Medicine, University of Southampton, Southampton, UK

Phone: +44 (023) 8120 6107. E-mail: [mluisamoro@yahoo.it](mailto:mluisamoro@yahoo.it) or [d.boche@soton.ac.uk](mailto:d.boche@soton.ac.uk)

**KEY WORDS:**

**IsoAspartate, pyroglutamate, Alzheimer’s disease, amyloid-beta, ageing.**

**Table S1**: Characteristics of the old control and AD cohorts

1. Old controls

| **MRC ID** | **Ctrl brain bank ID** | **Gender** | **Age at death** | **APOE status** | **Braak stage** | **CERAD score** | **Thal phases** |
| --- | --- | --- | --- | --- | --- | --- | --- |
| BBN_8671 | 36 | F | 78 | 3.3 | II | n/a | n/a |
| BBN_8684 | 50 | M | 71 | 3.3 | I | n/a | n/a |
| BBN_8691 | 57 | F | 82 | 2.3 | III | n/a | n/a |
| BBN_8706 | 72 | M | 72 | 3.3 | I | n/a | n/a |
| BBN_8708 | 74 | M | 90 | 2.3 | II | n/a | n/a |
| BBN_8709 | 75 | M | 83 | 3.3 | II | n/a | n/a |
| BBN_8728 | 98 | F | 88 | 3.3 | II | n/a | n/a |
| BBN_8732 | 103 | F | 76 | 3.3 | II | n/a | n/a |
| BBN_8735 | 106 | F | 88 | 3.3 | 0 | n/a | n/a |
| BBN_8739 | 110 | F | 93 | 3.3 | II | n/a | n/a |
| BBN_8751 | 122 | M | 82 | 3.3 | II | n/a | n/a |
| BBN_8770 | 141 | F | 89 | 3.3 | II | n/a | n/a |
| BBN_8818 | 189 | F | 93 | 3.3 | I | n/a | n/a |
| BBN_8835 | 206 | F | 73 | 3.3 | I | n/a | n/a |
| BBN_8888 | 259 | M | 89 | 3.4 | II | n/a | n/a |
| BBN_8898 | 269 | F | 83 | 3.3 | II | n/a | n/a |
| BBN_8923 | 295 | M | 82 | 3.3 | II | n/a | n/a |
| BBN_8964 | 336 | F | 82 | 4.4 | II | n/a | n/a |
| BBN_9292 | 714 | M | 73 | 3.3 | III | 1 | 0 |
| BBN_9329 | 751 | M | 80 | 3.3 | 0 | 0 | n/a |
| BBN_9331 | 753 | M | 97 | 3.4 | IV | 1 | n/a |
| BBN_9340 | 762 | F | 94 | 2.3 | II | 0 | 0 |
| BBN_9344 | 766 | M | 92 | 3.4 | II | 1 | 2 |
| BBN_4205 | 781 | M | 87 | 3.3 | II | 0 | 0 |
| BBN_9354 | 786 | M | 85 | 3.3 | II | 1 | 2 |
| BBN_9359 | 803 | M | 77 | 3.3 | I | 1 | n/a |
| BBN_4229 | 818 | F | 87 | 2.3 | III |  | n/a |
| BBN_9365 | 826 | F | 86 | 3.4 | II | 0 | n/a |
| BBN_9392 | 854 | F | 96 | n/a | II | 1 | n/a |
| BBN_9407 | 870 | F | 90 | n/a | II | 1 | n/a |
| BBN_9408 | 871 | M | 87 | n/a | II | 1 | n/a |

1. AD cases

| **MRC ID** | **AD brain bank ID** | **Gender** | **Age at death** | **APOE status** | **Braak stage** | **CERAD score** | **Thal phases** | **Dementia duration (years)** |
| --- | --- | --- | --- | --- | --- | --- | --- | --- |
| BBN_8925 | 297 | M | 88 | 3.4 | VI | n/a | n/a | 8 |
| BBN_8997 | 370 | F | 74 | 3.4 | VI | n/a | n/a | 7 |
| BBN_9031 | 405 | M | 85 | 3.4 | VI | n/a | n/a | n/a |
| BBN_9076 | 451 | F | 84 | 3.4 | V | n/a | n/a | 10 |
| BBN_9078 | 453 | F | 78 | 4.4 | IV | n/a | n/a | 3 |
| BBN_9113 | 489 | F | 81 | 3.4 | V | n/a | n/a | n/a |
| BBN_9119 | 495 | M | 80 | 3.4 | V | n/a | n/a | 6 |
| BBN_9134 | 510 | M | 71 | 3.4 | V | n/a | n/a | 7 |
| BBN_9162 | 538 | M | 63 | 3.3 | VI | n/a | n/a | 5 |
| BBN_9179 | 558 | M | 64 | 3.4 | VI | n/a | n/a | n/a |
| BBN_9181 | 560 | F | 80 | 4.4 | V | n/a | n/a | 9 |
| BBN_9182 | 561 | M | 74 | 3.4 | V | n/a | n/a | n/a |
| BBN_9183 | 562 | F | 85 | 3.4 | V | n/a | n/a | n/a |
| BBN_9186 | 565 | F | 75 | 3.4 | VI | n/a | n/a | 6 |
| BBN_9188 | 567 | F | 68 | 3.4 | VI | n/a | n/a | 8 |
| BBN_9189 | 568 | F | 78 | 4.4 | VI | n/a | n/a | 17 |
| BBN_9221 | 601 | M | 68 | 3.3 | VI | n/a | n/a | 10 |
| BBN_9246 | 668 | F | 71 | 4.4 | VI | n/a | n/a | 15 |
| BBN_9252 | 674 | M | 81 | n/a | VI | n/a | n/a | 3 |
| BBN_9257 | 679 | F | 82 | 34 | IV | n/a | n/a | 5 |
| BBN_9261 | 683 | M | 83 | 3.3 | V | 3 | n/a | 5 |
| BBN_9263 | 685 | M | 74 | 2.3 | V | 2 | n/a | 10 |
| BBN_9281 | 703 | F | 83 | 3.4 | V | 2 | 4 | 9 |
| BBN_9283 | 705 | F | 82 | 2.4 | V | 3 | 4 | 10 |
| BBN_9323 | 745 | F | 84 | 2.3 | VI | 3 | 4 | 15 |
| BBN_9341 | 763 | F | 80 | 4.4 | V | 3 | 4 | 9 |
| BBN_9343 | 765 | M | 80 | 3.4 | IV | 2 | 4 | 3 |

n/a: non-available; Ctrl: control; AD: Alzheimer’s disease

**Figure S1**

OC+AD

**
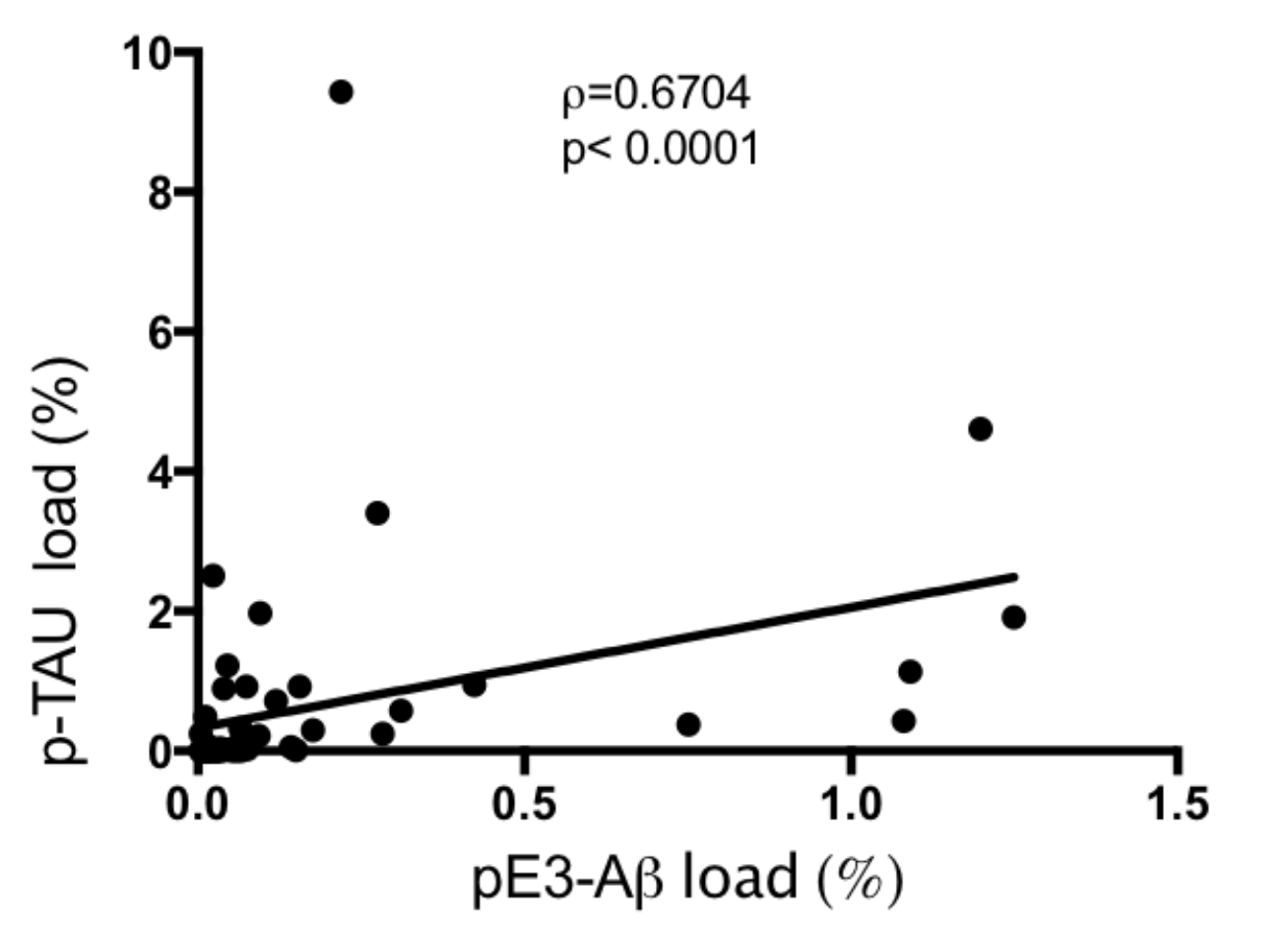
**

**Legend of Fig. S1**

Representation of the correlations between pE3-Aβ and phospho-TAU in OC and AD groups together.

The relationship between pE3-Aβ and p-TAU in OC and AD groups has been analysed according to the method described by Mandler M et al [1]*,* namely combining the data from AD and OC cohorts together and using the Spearman correlation coefficient, as determined by the normality of the markers. The threshold for statistical significance was set at 5%, and was determined by the use of SPSS 21.0 (IBM, US). A strong, positive correlation was identified between p-TAU and pE3-Aβ (ρ=0.6704, p<0.0001) in OC and AD groups together.

**Reference**

1.Mandler M, Walker L, Santic R, Hanson P, Upadhaya AR, Colloby SJ, Morris CM, Thal DR, Thomas AJ, Schneeberger A et al (2014) Pyroglutamylated amyloid-beta is associated with hyperphosphorylated tau and severity of Alzheimer's disease. Acta neuropathologica 128: 67-79 Doi 10.1007/s00401-014-1296-*9*
